# Diagnostic accuracy of high-risk HPV genotyping in women with high-grade cervical lesions: evidence for improving the cervical cancer screening strategy in China

**DOI:** 10.18632/oncotarget.11959

**Published:** 2016-09-10

**Authors:** Huihui Xu, Aifen Lin, Xiujuan Shao, Weiwu Shi, Yang Zhang, Weihua Yan

**Affiliations:** ^1^ Medical Research Center, Taizhou Hospital of Zhejiang Province, Wenzhou Medical University, Linhai, Zhejiang, China; ^2^ Human Tissue Bank, Taizhou Hospital of Zhejiang Province, Wenzhou Medical University, Linhai, Zhejiang, China; ^3^ Department of Gynecology, Taizhou Hospital of Zhejiang Province, Wenzhou Medical University, Linhai, Zhejiang, China

**Keywords:** high-risk human papillomavirus, genotyping, diagnosis, high-grade cervical lesion, cervical cancer screening

## Abstract

Currently, clinical data for primary HPV screening alone are lacking in China. Here, we evaluate cervical cancer screening with primary HPV genotyping, as well as possible future screening strategy. Overall, high-risk HPV (hrHPV) prevalence was 18.2% among hospital-based population in Taizhou area. For cervical intraepithelial neoplasia 2 or worse (CIN2+), the sensitivity of primary hrHPV genotyping strategy and current cervical cancer screening strategy were 93.5%, and 71.1%, respectively; whereas the specificity was 17.5%, and 62.4%, respectively. Current cervical screening strategy had slightly higher positive predictive values (28.4%) for CIN2+ than hrHPV genotyping strategy (21.9%), whereas primary hrHPV genotyping strategy demonstrated higher negative predictive values (94.7%) than current cervical screening strategy (91.1%). Compared to HPV35/39/45/51/56/59/66/68 genotypes, the odds ratios (OR) for CIN2+ in HPV16/18/31/33/52/58 infection women were 3.2 (95% confidence interval [CI] 2.3-4.1). Primary hrHPV genotyping strategy provides a better predictive value than HPV16/18 genotyping alone in guiding the clinical management of the current cervical cancer screening. HPV testing without adjunctive cytology may be sufficiently sensitive for primary cervical cancer screening.

## INTRODUCTION

Worldwide, cervical cancer is the second most common female malignancy. Approximately 500,000 new cases of cervical cancer are diagnosed and 275,000 deaths from cervical cancer occur annually. Persistent infection of high-risk human papillomavirus (hrHPV) is necessary for the development of high-grade intraepithelial neoplasia (CIN2/3) and cervical cancer [1]. More than 100 types of HPV can infect the anogenital epithelium, of which at least 14 types are classified as high-risk because of their strong carcinogenic potentials; sexually transmitted HPV may lead to cervical carcinogenesis [2]. HPV16 and HPV18 are the two most carcinogenic genotypes, accounting for 55-60% and 10-15% of cervical cancers, respectively. Additionally, HPV31, 33, 35, 39, 45, 51, 52, 56, 58, 59, 66, and 68 are also closely associated with cervical cancer. Significantly, epidemiologic studies have shown that nearly 100% of patients with cervical cancer test positive for HPV.

Public health screening programmes have successfully decreased cervical cancer incidence and mortality, including cervical cancer screenings and HPV vaccinations. Current guidelines for cervical cancer screening which cosponsored by the American Cancer Society (ACS), the American Society for Colposcopy and Cervical Pathology (ASCCP), and the American Society for Clinical Pathology (ASCP) in 2012, were recommendations address age-appropriate screening strategies, including the use of cytology and hrHPV testing (co-testing) [3, 4]. Recently, American Food and Drug Administration (FDA) approval hrHPV testing as an option for primary screening, which use of HPV16/18 genotyping along with a cocktail test of 12 other hrHPV genotypes [5]. However, genotyping solely for HPV16/18 would miss the majority of patients with low-grade squamous intraepithelial lesion (LSIL) who progress to high-grade cervical lesions [6]. In addition, because of the differences between the European/United States populations in terms of the screening frequency, HPV genotypic distribution, and HPV vaccination rates, the data collected from these countries may not represent the situation in China.

With the aim to establish a foundation for primary HPV screening in a certain area, and to support the local vaccination program in Taizhou region. This population-based, prospective observational study was designed to analyze the distribution of individual hrHPV genotypes across the complete spectrum of cervical disease; we have performed the primary HPV screening in detecting precancerous high-grade cervical lesions and cervical cancer.

## MATERIALS AND METHODS

### Study population

The Taizhou Area HPV study is a population-based, prospective observational study. We used HPV genotyping for primary cervical screening strategy, women with screen results of hrHPV positive referred directly to colposcopy biopsy. Moreover, the current cervical screening strategy with cytology and hrHPV testing (co-testing), the management of screen results stratified follow by: 1) atypical squamous cells of undetermined significance (ASCUS) or worse, referred directly to colposcopy biopsy; 2) cytology normal and HPV16/18 positive, referred directly to colposcopy biopsy [4, 7].

**Figure 1:**
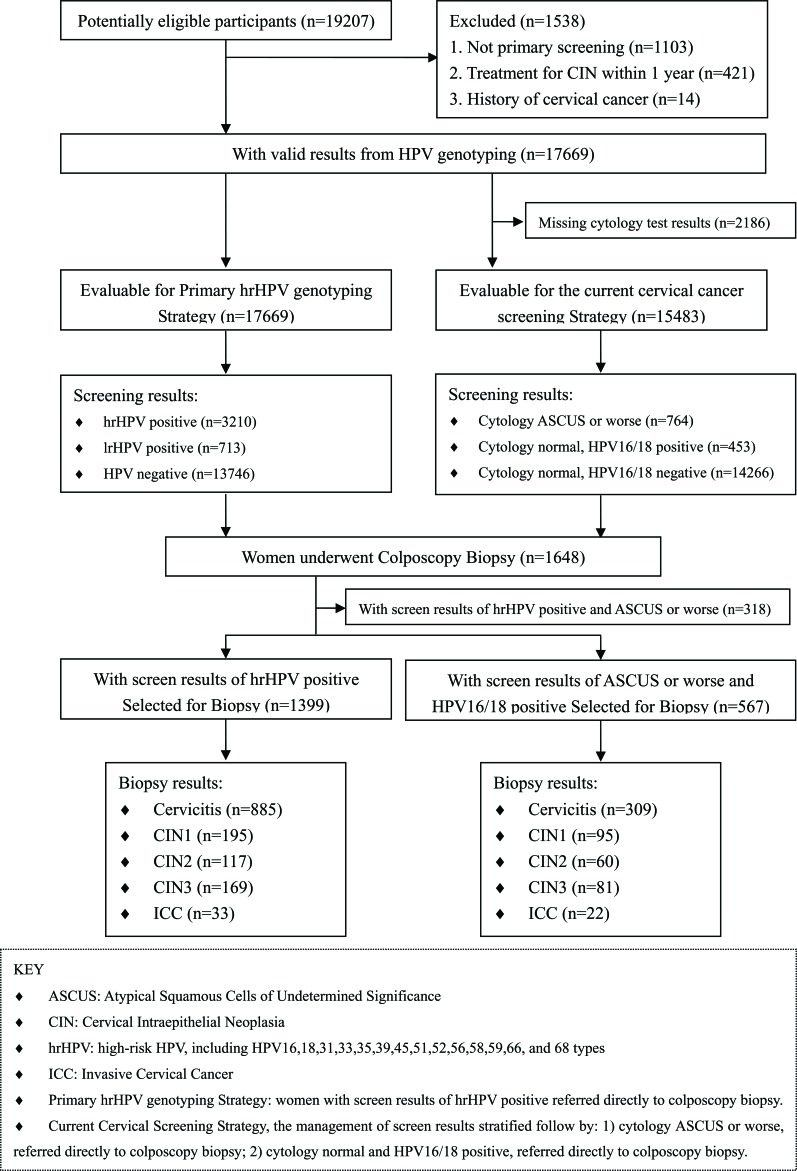
Flow chart of inclusion and exclusion criteria of the study population

Between December 2012 and April 2015, a total of 19207 consecutive women (median age 41.3 years; range 16-89) underwent cervical cancer screening in gynecological clinic at Taizhou Hospital of Zhejiang Province. The flowchart of study population was shown on Figure 1. Our final sample of 1648 women underwent colposcopy biopsy within 12 weeks. The study excluded hysterectomy, a history of cervical cancer, no treatment for CIN in the preceding 12 months or infection with HIV. Informed consent was obtained from participants in the study. For those participants younger than 18 years old, the consent form was signed by the parents of each participant.

### HPV genotyping

HPV genotyping was performed using the GP5+/bioGP6+-PCR/MPG assay, which was approved by the China Food and Drug Administration (CFDA Certified NO. (2014): 3400847). DNA was extracted from 200μl of each sample and collected in 50μl elution buffer according to the manufacturer's instructions. 5μl of extracted DNA was amplified by the GP5+/bioGP6+ broad-spectrum primer set with a final volume of 20μl. Reactions were heated for 5min at 95°C, followed by 35 repeated cycles of 94°C for 30s, 55°C for 30s, 72°C for 30s, and a final extension step at 72°C for 10min. Following the PCR amplification, 3μl of biotin-labeled PCR products and 22μl of hybridization solution containing 27 types of coupled beads of each set were transferred to 96-well plates. Hybridization was performed at 95°C for 5min followed by 48°C for 30min. Subsequently, streptavidin-phycoerythrin was added to each well at 48°C for 15min. The biotin-labeled PCR products were captured by HPV type-specific probes attached to color-coded beads, streptavidin-phycoerythrin was used as the reporter bound to the target, and the HPV genotypes were analyzed using the Luminex200^TM^ analyzer.

In short, it comprises the GP5+/bioGP6+-PCR, which using sets of biotinylated amplimers and a multiplex human papillomavirus genotyping (MPG) methods with bead-based Luminex suspension array technology [8, 9], which is able to simultaneously identify 14 hrHPV types including 16, 18, 31, 33, 35, 39, 45, 51, 52, 56, 58, 59, 66, 68 and 12 low-risk HPV (lrHPV) types including 6, 11, 26, 40, 42, 44, 53, 55, 61, 73, 82, 83 and β-globin gene (internal control).

### Diagnostic procedure

Cytological results, which blinded to the outcomes of HPV testing, were reported according to the 2001 Bethesda system. Cytological results were grouped as negative for intraepithelial lesion or malignancy (NILM), ASCUS, atypical squamous cells and cannot exclude high-grade squamous intraepithelial lesions (ASC-H), low-grade squamous intraepithelial lesions (LSIL), high-grade squamous intraepithelial lesions (HSIL), squamous cell carcinoma, atypical glandular cells (AGC), endocervical adenocarcinoma *in situ* (AIS), and adenocarcinoma.

Histopathologic diagnoses were adjudicated by pathologists and classified as normal, CIN grade 1, 2, 3 or invasive cervical cancer, according to international criteria. The suffix “+” means the indicated histology or more severe. Disease end points were histopathologically confirmed CIN2+ or CIN3+.

### Statistical analysis

Performance characteristics of hrHPV test (sensitivity, specificity, positive predictive value, negative predictive value) for identification of CIN2+ (to include CIN2, CIN3, adenocarcinoma *in situ*, and ICC) were determined using standard statistical tests. The chi-squared and Fisher's exact tests were used to evaluate relative CIN2+ risk associate with HPV genotypes, odds ratios (ORs) and relative 95% confidence interval (CI). All statistical analyses were performed using SPSS version 15.0 (SPSS Inc., Chicago, IL). *P* values were two-sided, and statistical significance was accepted if the *P* value was 0.05 or less.

## RESULTS

### Characteristics of the population

As shown in Table 1 and Figure 1, our final sample of 1648 women had diagnosed with biopsy, including 1081 women who diagnosed with cervicitis, 226 with CIN1, 122 with CIN2, 186 with CIN3, and 33 with ICC, respectively. The average ages of women with cervicitis, CIN1, CIN2, CIN3, and ICC were 42.1±10.5, 40.6±11.0, 41.4±9.1, 43.9±8.9, and 51.3±9.9, respectively. Women with cervical cancer were significantly older than those in the other groups (*P* < 0.001). According our recommended hrHPV genotyping for primary cervical screening strategy, 1399 women underwent colposcopy biopsy. According the current cervical screening strategy, 567 women underwent colposcopy biopsy.

**Table 1:** Clinical characteristics of the study population (*n* = 1648)

Characteristic	Cervicitis (*n* = 1081)	CIN1 (*n* = 226)	CIN2 (*n* = 122)	CIN3 (*n* = 186)	Cervical cancer (*n* = 33)
Age	**42.1±10.5**	**40.6±11.0**	**41.4±9.1**	**43.9±8.9**	51.3±9.9*
Primary hrHPV Genotyping Strategy	885	195	117	169	33
Current Cervical Screening Strategy	309	95	60	81	22

Abbreviations: CIN, cervical intraepithelial neoplasia; hrHPV, high-risk human papillomavirus.

* *P*<0.05 *vs*. each other groups.

### Prevalence of HPV genotypes

The overall prevalence of HPV was 22.2% (95% CI 21.6-22.8%), hrHPV and lrHPV infection rates were 18.2% (95% CI 17.6-18.7%) and 4.0% (95% CI 3.7-4.3%), respectively. Overall, HPV52 was the most prevalent genotype (4.9%), either alone or in combination with other types, followed by 16 (3.1%), 58 (2.7%), 39 (1.6%), 18 (1.5%), 56 (1.5%) (Table S1).

**Figure 2:**
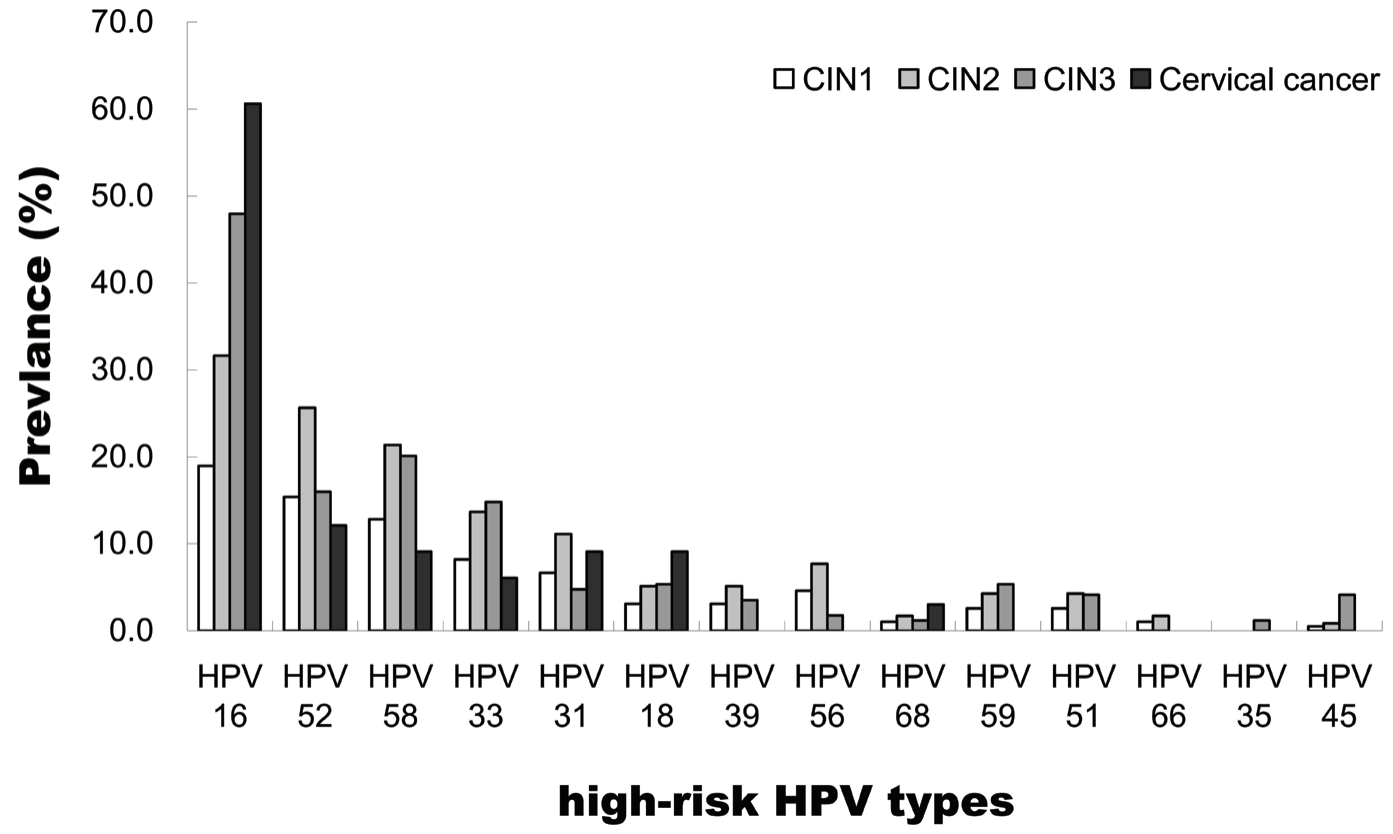
Relative distribution of high-risk HPV genotypes among HPV-positive cervical intraepithelial neoplasia (CIN; graded from 1 to 3) and cervical cancer women Women with multiple HPV types detected are counted to each type, and therefore counted more than once.

To assess the clinical predictive value for different hrHPV types, we further evaluated our data on the prevalence of individual hrHPV infection rates for cervical pathology status (Table 2 and Figure 2). For the patients with cervicitis/CIN1, HPV52 was the most common HPV type with the prevalence of 27.0%, followed by 58 (15.5%), 16 (14.4%), 39 (9.4%), 56 (9.0%) and 18 (8.4%). For the patients with CIN2+, HPV16 was the most common HPV type with the prevalence of 43.3%, followed by 58 (19.4%), 52 (19.1%), 33 (13.5%), 31 (7.5%) and 18 (5.6%). HPV16, 52 and 58 were the three HPV types most commonly found in any cervical pathology status. Notably, HPV52 was the most common type among women with cervicitis/CIN1, but the distribution changed remarkably for CIN2+, where ranked the third.

**Table 2:** Prevalence rates of hrHPV genotypes for cervical pathology status among hrHPV-positive women (*n* = 1399)

hrHPV genotypes	Cervical cancer (*n* = 33)		CIN3 (*n* = 169)		CIN2 (*n* = 117)		CIN1 (*n* = 195)			Cervicitis (*n* = 885)
	*n*	%(95%CI)	*n*	%(95%CI)	*n*	%(95%CI)	*n*	%(95%CI)	*n*	%(95%CI)
HPV 52	4	12.1(1.0-23.3)	27	16.0(10.5-21.5)	30	25.6(17.7-33.6)	57	29.2(22.8-35.6)	235	26.6(23.6-29.5)
HPV 16	20	60.6(43.9-77.3)	81	47.9(40.4-55.5)	37	31.6(23.2-40.0)	33	16.9(11.7-22.2)	123	13.9(11.6-16.2)
HPV 58	3	9.1(0.0-18.9)	34	20.1(14.1-26.2)	25	21.4(13.9-28.8)	28	14.4(9.4-19.3)	139	15.7(13.3-18.1)
HPV 39	0	0.0(0.0-0.0)	6	3.6(0.8-6.3)	6	5.1(1.1-9.1)	14	7.2(3.6-10.8)	88	9.9(8.0-11.9)
HPV 56	0	0.0(0.0-0.0)	3	1.8(0.0-3.8)	9	7.7(2.9-12.5)	14	7.2(3.6-10.8)	83	9.4(7.5-11.3)
HPV 18	3	9.1(0.0-18.9)	9	5.3(1.9-8.7)	6	5.1(1.1-9.1)	16	8.2(4.4-12.1)	75	8.5(6.6-10.3)
HPV 68	1	3.0(0.0-8.9)	2	1.2(0.0-2.8)	2	1.7(0.0-4.1)	9	4.6(1.7-7.6)	77	8.7(6.8-10.6)
HPV 33	2	6.1(0.0-14.2)	25	14.8(9.4-20.1)	16	13.7(7.4-19.9)	14	7.2(3.6-10.8)	57	6.4(4.8-8.1)
HPV 59	0	0.0(0.0-0.0)	9	5.3(1.9-8.7)	5	4.3(0.6-7.9)	19	9.7(5.6-13.9)	55	6.2(4.6-7.8)
HPV 51	0	0.0(0.0-0.0)	7	4.1(1.1-7.1)	5	4.3(0.6-7.9)	11	5.6(2.4-8.9)	44	5.0(3.5-6.4)
HPV 31	3	9.1(0.0-18.9)	8	4.7(1.5-7.9)	13	11.1(5.4-16.8)	13	6.7(3.2-10.2)	35	4.0(2.7-5.2)
HPV 66	0	0.0(0.0-0.0)	0	0.0(0.0-0.0)	2	1.7(0.0-4.1)	9	4.6(1.7-7.6)	38	4.3(3.0-5.6)
HPV 35	0	0.0(0.0-0.0)	2	1.2(0.0-2.8)	0	0.0(0.0-0.0)	2	1.0(0.0-2.4)	16	1.8(0.9-2.7)
HPV 45	0	0.0(0.0-0.0)	7	4.1(1.1-7.1)	1	0.9(0.0-2.5)	2	1.0(0.0-2.4)	15	1.7(0.8-2.5)

Abbreviations: CIN, cervical intraepithelial neoplasia; hrHPV, high-risk human papillomavirus.

* Women with multiple HPV types detected are counted to each type, and therefore counted more than once.

### Primary hrHPV genotyping strategy

In this study, we recommended hrHPV genotyping for primary cervical screening strategy, when women with screen results of hrHPV positive referred directly to biopsy. According to this guideline, 3210 women need biopsy, irrespective of women age. In fact, 1399 (43.6%) women with hrHPV infection accepted biopsy, including 885 women with cervicitis, 195 with CIN1, 117 with CIN2, 169 with CIN3, and 33 with ICC, respectively. According this screening strategy, the sensitivity and specificity for detecting CIN2+ were 93.5 % and 17.5%, respectively. The positive predictive value and negative predictive value were 22.8% and 91.2%, respectively.

According to the current cervical screening strategy, 1217 women need biopsy, irrespective of women age. In fact, 567 (46.6%) women with ASCUS+ or HPV16/18 positive accepted biopsy, including 309 women with cervicitis, 95 with CIN1, 60 with CIN2, 81 with CIN3, and 22 with ICC, respectively. The sensitivity and specificity for detecting CIN2+ were 71.1% and 62.4%, respectively. The positive predictive value and negative predictive value were 28.4% and 91.1%, respectively. Compared to the current cervical screening, primary hrHPV genotyping test had higher sensitivity and lower specificity (Table 3).

**Table 3:** The accuracy values of different triage strategies for the detection of CIN2+/CIN3+

Screen Strategy	Performance measure (95% CI)	CIN2+	CIN3+
Current cervical cancer screening	Sensitivity	71.1(65.2-77.0)	71.4(63.9-78.9)
	Specificity	62.4(59.5-65.3)	60.0(57.2-62.8)
	Positive predictive value	28.4(24.6-32.1)	17.7(14.6-20.9)
	Negative predictive value	91.2(89.1-93.2)	94.6(92.9-96.2)
Primary hrHPV genotyping test	Sensitivity	93.5(90.9-96.2)	92.2(88.7-95.8)
	Specificity	17.5(15.3-19.4)	17.8(15.7-19.8)
	Positive predictive value	22.8(20.6-25.0)	14.4(12.6-16.3)
	Negative predictive value	91.2(87.6-94.7)	93.2(90.0-96.3)

Abbreviations: CIN, cervical intraepithelial neoplasia; hrHPV, high-risk human papillomavirus; CI: 95% confidence interval.

### Management of women with hrHPV infection

We further examined whether hrHPV positive women for HPV16/18 (2 types), or HPV16/18/31/33/52/58 (the 6 major carcinogenic types) can serve as a triage tool to discriminate women who need biopsy immediately. Compared to 12 other hrHPV infection women, the ORs for CIN2+ in HPV16/18 (2 types) infection women was 2.7 (95%CI 2.1-3.4). Notably, the relative CIN2+ risk (ORs) was 3.2(95%CI 2.3-4.1) for women with HPV16/18/31/33/52/58 (6 types) infection compared to women with HPV35/39/45/51/56/59/66/68 infection. For detecting CIN2+, the sensitivity and specificity were 86.5% and 43.5%, respectively. The positive predictive value and negative predictive value were 28.5% and 92.5%, respectively. Therefore, when women with HPV16/18/31/33/52/58 infection can be recommended colposcopy biopsy immediately.

Women who are HPV35/39/45/51/56/59/66/68 positive should be detected with cervical cytological testing. Among women with ASCUS cytology, HPV35/39/45/51/56/59/66/68 infection rate was 32.4%. Women with ASCUS+ can be recommended colposcopy biopsy immediately. The accuracy values of different triage strategies for the detection of CIN2+/CIN3+ were shown in Table S2.

## DISCUSSION

In recent years, hrHPV testing for triaging ASCUS and co-testing with cervical cytology have been implemented in clinical practice [4, 10]. However, clinical data for primary HPV screening alone are currently lacking [5, 11]. Numerous of prospective randomized screening trials, primarily from Europe or United States, have documented that co-testing offers minimal increased protection against the subsequent progression of cervical lesions compared to primary HPV testing, which is more sensitive than cytology screening, but specificity depends on subsequent evaluation strategies and screening frequencies [10–13]. Recently, American FDA approval HPV testing as an option for primary screening [5], however, clinical practice guidelines for primary HPV screening strategy, which do not exist in China. It is important to improve knowledge about the complete carcinogenic process for individual hrHPV genotypes from infection to cervical cancer, which may serve as predictive markers of disease persistence and progression. Therefore, our study addressed an important question is to detect hrHPV genotypes initial cytology specimens of whether associated with the progression of high-grade cervical lesions during pathology diagnose, in order to reduce the number of biopsy and improve the CIN2+ detection rate, further renewed the cervical cancer screening strategies in China.

The prevalence of hrHPV (18.2%) obtained in this present study were similar to that in Hangzhou (19.9%) and Nanchang (18.4%) which also region in southeast of China [14]. Consistent with the data generated by Chinese population-based investigations, HPV16, HPV52, and HPV58 were found to be the dominant hrHPV types [14, 15], but unlike in a meta-analysis that summarized global reports [16] in which HPV16, 18, and 45, HPV16, 18, and 33 or HPV16, 18, and 58 were most commonly detected. In our population, HPV52 and HPV58 accounted for 26.4%, which are all common among Asian populations and markedly higher than the global rate of 14.0% [16]. HPV52 was detected in 26.6%, 29.2%, and 19.1% of women with cervicitis, CIN1, and CIN2+, respectively. HPV58 was detected in 15.7%, 14.4%, and 19.4% of women with cervicitis, CIN1, and CIN2+, respectively. These data showed that HPV52 is more common among cervicitis women, whereas HPV58 is more common among CIN2+ women, which was also confirmed by several other studies [16–19]. HPV58, which is associated with a higher risk of developing high-grade cervical lesions than other non-HPV16 types [20], has been found in a relatively higher proportion of women with high-grade cervical lesions in Eastern Asia than elsewhere [16, 21].

In our population, we found that HPV16, 31, 33, and 58 increased the risk for CIN1 lesions progress to CIN2 or worse (Figure 2). HPV genotyping test will enable us to characterize a woman's cervical disease risk more precisely, the OR for CIN2+ in HPV16/18/31/33/52/58 positive women was 3.2 (95%CI 2.3-4.1) when compared to HPV35/39/45/51/56/59/66/68 positive women. Consistent with the data generated by global meta-analysis, HPV16/18/31/33/52/58 are the six most common genotypes detected in women with cervical cancer worldwide, according for > 90% of cervical cancer in each area [16, 22–24]. In 2015, the Advisory Committee on Immunization Practices (ACIP) recommended 9-valent HPV vaccine (9vHPV) which contains HPV6, 11, 16, 18, 31, 33, 45, 52, and 58 virus-like particle (VLP) was licensed by the FDA [25, 26]. The 9vHPV vaccine covers the 6 major carcinogenic HPV genotypes which prevalent approximately 70% of hrHPV infection in Taizhou area. These findings defined principles for the national population-based screening programs and vaccination in southeast China.

Compared with current cervical cancer screening strategy, we recommended screening strategy had higher sensitivity (93.5%) and higher negative predictive values (91.2%). In the present study, the current cervical screening strategy had been missing the majority of women (178 cases, 52.2%) with hrHPV infections who progress to high-grade cervical lesions (CIN2+) (Table 1). Our results supported that HPV16/18/31/33/52/58-positive women need immediate biopsy, which would increase the number of CIN2+ by approximately doubling. In a 14-year follow-up of a randomized primary HPV screening, HPV16/18/31/33/45/52/58 had 14-year cumulative incidences 73.9% of CIN2+ and all hrHPV genotypes contributed 86.9% [27], and 30% of cervical cancers are associated with hrHPV genotypes other than HPV16 and HPV18 [28]. In addition, it has been reported that the reassurance of HPV-negative women with primary HPV screening every 3 years was nearly equivalent to co-testing every 5 years [12]. The 18-year follow-up analysis showed that hrHPV-positive women were more likely to be diagnosed with CIN2+ (*P* < 0.001) 10-18 years after enrollment compared with hrHPV-negative women. The 18-year cumulative incidence rates (CIRs) of CIN2+ among hrHPV-positive and hrHPV-negative women were 23.2% and 1.5%, respectively. [29]. These findings support the hrHPV genotyping for primary cervical screening strategy could replace co-testing.

However, poor specificity (17.5%) and poor positive predictive value (22.8%) for the determination of CIN2+ in the current study would be limits the use of our recommended screening strategy. In our study, women with HPV16/18/31/33/52/58 (the 6 major carcinogenic types) infections rate were 71.1% (2282/3210) of overall hrHPV-positive women. In order to reduce the number of biopsy, we suggested when women with HPV16/18/31/33/52/58 infection can be recommended colposcopy biopsy immediately. For detecting CIN2+, the specificity and positive predictive value were 43.5% and 28.5%, respectively. In order to improve the CIN2+ detection rate, our data suggested that reflex cytology for women with HPV35/39/45/51/56/59/66/68 infection will be clinically useful as a triage test tool for immediate biopsy for women with ASCUS or worse.

In summary, hrHPV genotyping provide a better predictive value than HPV16/18 genotyping alone in guiding the clinical management of the current cervical cancer screening. HPV testing without adjunctive cytology may be sufficiently sensitive for primary cervical cancer screening.

## SUPPLEMENTARY MATERIALS




