# Supplementary material for: Diagnostic accuracy of high-risk HPV genotyping in women with high-grade cervical lesions: evidence for improving the cervical cancer screening strategy in China
Source: Oncotarget. 2016 Sep 10;7(50):83775–83. doi: 10.18632/oncotarget.11959 (PMC5347804; doi:10.18632/oncotarget.11959)
Supplement: Supplementary file 1 [file oncotarget-07-83775-s001.doc]

| Table S1. Prevalence and genotypes distribution of HPV by cytological outcomes among Chinese women (Taizhou, Zhejiang, 2015)   | **HPV Status*** | **HPV test**  **(n=17669)** | **Cytology test ( n=15483)** | | | --- | --- | --- | --- | | **NILM**  **(n=14719)** | **ASCUS or worse**  **(n=764)** | | Negative | 13746(77.8) | 12078(82.1) | 219(28.7) | | Positive | 3923(22.2) | 2641(17.9) | 545(71.3) | |  |  |  |  | | hrHPV types |  |  |  | | HPV 52 | 871(4.9) | 596(4.0) | 111(14.5) | | HPV 16 | 545(3.1) | 290(2.0) | 114(14.9) | | HPV 58 | 483(2.7) | 281(1.9) | 97(12.7) | | HPV 39 | 278(1.6) | 209(1.4) | 28(3.7) | | HPV 18 | 264(1.5) | 179(1.2) | 34(4.5) | | HPV 56 | 257(1.5) | 165(1.1) | 43(5.6) | | HPV 68 | 250(1.4) | 178(1.2) | 28(3.7) | | HPV 33 | 230(1.3) | 141(1.0) | 45(5.9) | | HPV 59 | 188(1.1) | 136(0.9) | 20(2.6) | | HPV 51 | 157(0.9) | 104(0.7) | 25(3.3) | | HPV 31 | 159(0.9) | 85(0.6) | 38(5.0) | | HPV 66 | 117(0.7) | 81(0.6) | 16(2.1) | | HPV 35 | 60(0.3) | 39(0.3) | 8(1.0) | | HPV 45 | 61(0.3) | 38(0.3) | 7(0.9) | |  |  |  |  | | lrHPV types |  |  |  | | HPV 61 | 274(1.6) | 198(1.3) | 26(3.4) | | HPV 06 | 166(0.9) | 108(0.7) | 24(3.1) | | HPV 44 | 164(0.9) | 130(0.9) | 12(1.6) | | HPV 53 | 155(0.9) | 93(0.6) | 23(3.0) | | HPV 82 | 110(0.6) | 79(0.5) | 14(1.8) | | HPV 55 | 98(0.6) | 81(0.6) | 6(0.8) | | HPV 11 | 90(0.5) | 51(0.3) | 16(2.1) | | HPV 42 | 55(0.3) | 35(0.2) | 5(0.7) | | HPV 40 | 54(0.3) | 37(0.3) | 9(1.2) | | HPV 83 | 25(0.1) | 20(0.1) | 4(0.5) | | HPV 26 | 7(0.0) | 3(0.0) | 1(0.1) | | HPV 73 | 6(0.0) | 4(0.0) | 2(0.3) | | Abbreviations: ASCUS, atypical squamous cells of undetermined significance; NILM, negative for intraepithelial lesion or malignancy; hrHPV, high-risk human papillomavirus; lrHPV, low-risk human papillomavirus.  * Women with multiple HPV types detected are counted to each type, and therefore counted more than once. | | | | |
| --- | --- | --- | --- | --- | --- | --- | --- | --- | --- | --- | --- | --- | --- | --- | --- | --- | --- | --- | --- | --- | --- | --- | --- | --- | --- | --- | --- | --- | --- | --- | --- | --- | --- | --- | --- | --- | --- | --- | --- | --- | --- | --- | --- | --- | --- | --- | --- | --- | --- | --- | --- | --- | --- | --- | --- | --- | --- | --- | --- | --- | --- | --- | --- | --- | --- | --- | --- | --- | --- | --- | --- | --- | --- | --- | --- | --- | --- | --- | --- | --- | --- | --- | --- | --- | --- | --- | --- | --- | --- | --- | --- | --- | --- | --- | --- | --- | --- | --- | --- | --- | --- | --- | --- | --- | --- | --- | --- | --- | --- | --- | --- | --- | --- | --- | --- | --- | --- | --- | --- | --- | --- | --- | --- | --- | --- | --- | --- | --- | --- | --- | --- | --- | --- | --- | --- | --- | --- | --- |
