# Supplementary material for: Diagnostic accuracy of high-risk HPV genotyping in women with high-grade cervical lesions: evidence for improving the cervical cancer screening strategy in China
Source: Oncotarget. 2016 Sep 10;7(50):83775–83. doi: 10.18632/oncotarget.11959 (PMC5347804; doi:10.18632/oncotarget.11959)
Supplement: Supplementary file 2 [file oncotarget-07-83775-s002.doc]

| Table S2. The accuracy values of different triage strategies for the detection of CIN2+/CIN3+ | | | |
| --- | --- | --- | --- |
| Screen Strategy | Accuracy values  (95% CI ) | CIN2+ | CIN3+ |
| **Primary hrHPV genotyping strategy** | |  |  |
| HPV16/18-positive | Sensitivity | 44.4(39.1-49.7) | 49.1(42.4-55.7) |
|  | Specificity | 81.8(79.7-83.9) | 80.3(78.2-82.3) |
|  | PPV | 38.8(34.0-43.7) | 27.5(23.1-31.9) |
|  | NPV | 85.0(83.0-87.0) | 91.2(89.6-92.7) |
|  |  |  |  |
| HPV16/18/31/33/  52/58-positive | Sensitivity | 86.5(82.8-90.1) | 86.7(82.2-91.2) |
|  | Specificity | 43.5(40.8-46.2) | 41.0(38.4-43.5) |
|  | PPV | 28.5(25.7-31.2) | 18.3(15.9-20.7) |
|  | NPV | 92.5(90.4-94.6) | 95.3(93.6-97.0) |
|  |  |  |  |
| HPV16/18/31/33/  35/39/45/51/52/56/  58/59/66/68-positive | Sensitivity | 93.5(90.9-96.2) | 92.2(88.7-95.8) |
|  | Specificity | 17.5(15.3-19.4) | 17.8(15.7-19.8) |
|  | PPV | 22.8(20.6-25.0) | 14.4(12.6-16.3) |
|  | NPV | 91.2(87.6-94.7) | 93.2(90.0-96.3) |
|  |  |  |  |
| ASCUS or worse and HPV16/18/31/33/  52/58-positive | Sensitivity | 91.6(87.9-95.2) | 90.7(85.9-95.5) |
|  | Specificity | 31.8(29.0-34.6) | 30.0(27.4-32.6) |
|  | PPV | 21.9(19.3-24.6) | 13.5(11.3-15.7) |
|  | NPV | 94.7(92.4-97.0) | 96.4(94.5-98.3) |
|  |  |  |  |
| **Current cervical cancer screening** | |  |  |
| ASCUS or worse | Sensitivity | 55.4(48.8-61.9) | 56.4(48.2-64.6) |
|  | Specificity | 77.6(75.1-80.1) | 75.3(72.9-77.8) |
|  | PPV | 34.0(29.1-38.8) | 21.6(17.4-25.9) |
|  | NPV | 89.3(87.3-91.3) | 93.5(91.9-95.1) |
|  |  |  |  |
| ASCUS or worse and HPV16/18-positive | Sensitivity | 71.1(65.2-77.0) | 71.4(63.9-78.9) |
|  | Specificity | 62.4(59.5-65.3) | 60.0(57.2-62.8) |
|  | PPV | 28.4(24.6-32.1) | 17.7(14.6-20.9) |
|  | NPV | 91.1(89.1-93.2) | 94.6(92.9-96.2) |

Abbreviations: CIN, cervical intraepithelial neoplasia; ASCUS, atypical squamous cells of undetermined significance; hrHPV, high-risk human papillomavirus; CI: 95% confidence interval; PPV, positive predictive value; NPV, negative predictive value.
